# Draft Genome Sequence of *Pseudomonas* sp. Strain ADP, a Bacterial Model for Studying the Degradation of the Herbicide Atrazine

**DOI:** 10.1128/genomeA.01733-15

**Published:** 2016-02-11

**Authors:** Marion Devers-Lamrani, Aymé Spor, Arnaud Mounier, Fabrice Martin-Laurent

**Affiliations:** INRA, UMR 1347 Agroécologie, Dijon, France

## Abstract

We report here the 7,259,392-bp draft genome of *Pseudomonas* sp. strain ADP. This is a bacterial strain that was first isolated in the 1990s from soil for its ability to mineralize the herbicide atrazine. It has extensively been studied as a model to understand the atrazine biodegradation pathway. This genome will be used as a reference and compared to evolved populations obtained by experimental evolution conducted on this strain under atrazine selection pressure.

## GENOME ANNOUNCEMENT

*Pseudomonas* sp. strain ADP was isolated in the 1990s from an atrazine spill site (Little Falls, MN, USA) for its ability to mineralize this herbicide (1, 2). In this strain, the *atz* genes involved in atrazine mineralization are located on a 108-kb plasmid, pADP-1 (3). A decade ago, we initiated an experiment on this strain to study the evolution of the atrazine degradation pathway under atrazine selection pressure. This evolution experiment gave rise to newly evolved populations displaying an improved capacity for atrazine degradation (4). To determine the genetic modifications that occurred during the course of evolution, genomes of the ancestral and evolved populations have been sequenced. Here, we describe the draft genome sequence of the ancestral strain of *Pseudomonas* sp. strain ADP originating from the laboratory of Larry Wackett and maintained in the lab for two decades.

Two DNA genomic libraries were constructed using the Nextera sequencing kits: a paired-end library (insert size of 500 bp), and a mate-pair library, with a theoretical insert size of 8 kb. They were bidirectionally sequenced using a MiSeq sequencer with 250-bp read chemistry (Illumina). The removal of low-quality reads (*Q* < 30) and trimming of low-quality read ends (*Q* < 30), adapters, and cloning vector sequences resulted in 4,421,329 mate-pair and 2,928,198 paired-end reads, with an average length of 237 bp. To optimize the assembly, the reads corresponding to pADP-1 (GenBank accession no. U66917) were separated from the ones corresponding to the chromosome by using a homemade script. *De novo* assembly and scaffolding of both sets of reads were performed using Velvet 1.2.10 (5). They were optimized by (i) using the Velvet estimation of the insert size of the mate pairs (6,000 ± 3,500 bp) and paired ends (450 ± 50 bp) and by (ii) choosing the k-mer value generating the higher *N*_50_ and the lower number of scaffolds.

For the chromosome, 22 scaffolds (>1,000 bp) containing 108 gaps were generated (147-bp k-mer, 46× coverage). The draft chromosome sequence has a total length of 7,151,428 bp, and the largest scaffold measures 5,153,685 bp, which corresponds to the *N*_50_. For the plasmid, a unique circular sequence of 107,964 bp with no gap was obtained (155-bp k-mer, 85× coverage). The G+C content was 66.8% for the chromosome and 62.6% for the plasmid. Gene prediction using RAST (6) revealed the presence of 6,437 open reading frames and 5 rRNA gene clusters. Based on 16S rRNA gene analysis, the closest neighbor was *Pseudomonas citronellolis* strain odb7 (99% similarity). Compared to the available pADP-1 sequence, the plasmid of strain ADP presents the same modular organization but some differences within the sequence (99% similarity). To determine the genetic basis of the evolution of the atrazine degradation capacity that occurred during our evolution experiment, further studies will aim to compare this genome to the ones of the evolved populations.

### Nucleotide sequence accession numbers.

This whole-genome shotgun project has been deposited at DDBJ/EMBL/GenBank under the accession no. LKAX00000000. The version described in this paper is version LKAX01000000.
